# Examining telehealth use among primary care patients, providers, and clinics during the COVID-19 pandemic

**DOI:** 10.1186/s12875-022-01738-3

**Published:** 2022-06-18

**Authors:** Claudia Der-Martirosian, Karen Chu, W. Neil Steers, Tamar Wyte-Lake, Michelle D. Balut, Aram Dobalian, Leonie Heyworth, Neil M. Paige, Lucinda Leung

**Affiliations:** 1 Veterans Emergency Management Evaluation Center (VEMEC), US Department of Veterans Affairs, North Hills, CA USA; 2 Center for the Study of Healthcare Innovation, Implementation & Policy (CSHIIP), US Department of Veterans Affairs, Greater Los Angeles Healthcare System (VAGLAHCS), Los Angeles, CA USA; 3 Division of Health Services Management and Policy, The Ohio State University College of Public Health, Columbus, OH USA; 4 Office of Connected Care, Veterans Health Administration, US Department of Veterans Affairs, Washington DC, USA; 5 Department of Medicine, University of California, San Diego, San Diego, CA USA; 6 US Department of Veterans Affairs, Greater Los Angeles Healthcare System (VAGLAHCS), Los Angeles, CA USA; 7 Division of General Internal Medicine Health Services Research at UCLA David Geffen School of Medicine, Los Angeles, CA USA

**Keywords:** Telehealth, Primary Care, Video-Based Care, Veterans, US Veterans Health Admininstration

## Abstract

**Background:**

At the onset of COVID-19, there was a rapid expansion of telehealth (video/telephone) visits to maintain delivery of primary care (PC) services at the Veterans Health Administration (VA). This study examines patient, provider, and site-level characteristics of any virtual and video-based care in PC.

**Methods:**

Interrupted time series (ITS) design was conducted using VA administrative/clinical, electronic healthcare data, 12-months before and 12-months after COVID-19 onset (set at March 2020) at the VA Greater Los Angeles Healthcare System (GLA), between 2019 and 2021. Patients with at least one visit to a VA PC clinic at GLA (*n* = 547,730 visits) were included in the analysis. The two main outcomes for this study were 1) any telehealth (versus in-person), as well as 2) video-based care (versus telephone). For the ITS analysis, segmented logistic regression on repeated monthly observations of any telehealth and video-based care was used.

**Results:**

Percent telehealth and video use increased from 13.9 to 63.1%, and 0.3 to 11.3%, respectively, before to after COVID-19 onset. According to adjusted percentages, GLA community-based clinics (37.7%, versus 29.8% in hospital-based clinics, *p* < .001), social workers/pharmacists/dietitians (53.7%, versus 34.0% for PC clinicians, *p* < .001), and minority groups, non-Hispanic African Americans (36.3%) and Hispanics (34.4%, versus 35.3% for Whites, *p* < .001) were more likely to use telephone than video. Conversely, mental health providers (43.3%) compared to PC clinicians (15.3%), and women (for all age groups, except 75+) compared to men, were more likely to use video than telephone (all *p*’s < .001).

**Conclusions:**

Since telehealth care provision is likely to continue after COVID-19, additional research is needed to identify which PC outpatient services are better suited for telephone (e.g., case management) versus video-based care (e.g., integrated mental health visits). Additionally, it is important to understand how all clinics can systematically increase access to both telephone- and video-based PC services, while ensuring equitable care for all patient populations.

## Introduction

With the onset of the COVID-19 pandemic, there was an urgent need to expand telehealth services in outpatient care, so care, albeit limited in some cases, could still be provided while keeping patients safe from the novel coronavirus [1, 2]. During the past 20 months, COVID-19 provided the opportunity and the push to increase the expansion of home-based virtual care in primary care (PC) [3–7]. Telehealth is broadly defined as using technology for a remote medical encounter [3]. Recent research on PC and telehealth services has expanded rapidly both within VA, as well as outside of VA, focusing on a wide variety of topics, such as identifying ways to integrate telehealth in PC [8, 9], patient satisfaction with telehealth services [10], organizational and external factors associated with video use [11], patient characteristics of telehealth use and disparities in access to video visits [6, 12, 13], among other topics. Like many healthcare settings, access to VA telehealth services, especially VA Video Connect (VVC) [6, 11], which is the primary approved platform for patient home-based video visits at the VA [14–19], increased dramatically immediately after onset of COVID-19 [4].

While the demand for patient home-based virtual visits in PC beyond the pandemic is still unknown, the provision of telehealth services in PC will continue after COVID-19. Now more than ever, virtual-based care has been integrated as another mode of care delivery across healthcare systems, including the Veterans Health Administration (VA). Although the increase in telehealth and video use in PC, both at the VA and outside of VA during COVID-19, is widely recognized [1, 2, 4–7, 11, 20–27], it is still unclear how the use of telehealth services in PC affects care at multiple levels (i.e., patient, provider, clinic). The VA is an ideal place to study telehealth service utilization, since it was an early adopter and a national leader [18] in telehealth, with over two decades of experience in virtual care [4]. Furthermore, the VA’s main hospitals are connected to surrounding community outpatient centers, which are often located in less urban/rural areas, with limited cellular and internet bandwidth. Moreover, the VA’s team-based PC model, which is patient-aligned care teams (PACT) with interdisciplinary providers, are patient-centered medical models similar to various non-VA settings.

In a recent study [28], a mixed methods approach was used to examine the implementation of telehealth services during COVID-19 at three clinics (PC, Cardiology, and Home-Based Primary Care) at one VA medical center, and the authors illustrated that the determinants of telehealth utilization were multifaceted; further examination of patient, provider, clinic, and site characteristics are now needed to better understand what factors are associated with telehealth use. To our knowledge, there are no studies that have simultaneously examined multi-level characteristics of telehealth utilization. This study focuses on the VA Greater Los Angeles Healthcare System (GLA), which includes a hospital-based clinic and surrounding large and small community-based clinics and examines the impact of provider type as well as site type on telehealth or video use in PC. In addition to provider and site characteristics, this study also focuses on patient characteristics, and examines differences in telehealth (or video) by sex and age, as well as by race/ethnicity, within the context of before and after the onset of the pandemic. The overarching research question for the study includes: what were the impacts of COVID onset (restriction of in-person services), re-expansion of in-person services, and the 2020 flu season on telehealth and video use? And, within this context, four main hypotheses were tested to examine patient, provider, and site-level characteristics of telehealth (or video) use: Patient: 1) younger women Veterans are more likely to use telehealth (or video) services compared to younger men Veterans, while 2) racial/minority groups are less likely to use telehealth (or video) compared to Whites; Provider: 3) specialty clinicians in primary care clinics (such as mental health providers and social workers) are more likely to use telehealth (or video) compared to primary care providers; Site: 4) the main medical facility is more likely to have telehealth (or video) visits compared to the community clinics.

## Methods

VA administrative/clinical, electronic healthcare data were used to examine the characteristics of any telehealth use, as well as video use in PC at GLA, during the 24-month study period (March 1, 2019, through February 28, 2021), which comprised an ITS with 12-months of dependent variable measurements before and 12-months after the onset of COVID-19 in March 2020. The study sample included a total of 547,730 PC visits: 299,881 PC visits (64,362 patients) during the 12-months before the onset of COVID-19, and 247,849 PC visits (48,729 patients) during the 12-months after the onset of COVID-19.

This study was part of a larger study that examined implementation of telehealth services at three types of clinics/programs (PC, HBPC, Cardiology) in GLA [28], and it was approved by the GLA Institutional Review Board (IRB #1616051–6).

### Measures

In this study, telehealth is further defined as synchronous communication between clinicians and patients (from home) using either audio (phone) or audio and video (video-based care) [3]. The study included two main dependent variables: 1) telehealth use (phone or video) vs. in-person care, among all PC users during the 24-month study period, and 2) video- vs. phone-based care, among all PC telehealth users during the 24-month study period. We included covariate measures at three levels: patient, provider, and site. Patient-level covariates known to be associated with telehealth use included: patient socio-demographics (age and sex interaction, race/ethnicity, marital status, health insurance) [6, 7, 23, 25] and Nosos comorbidity score (a cost-based risk adjustment scale used by VA) [29]. Four age categories (18–44, 45–64, 65–74, 75+), two categories for sex (male, female), five race/ethnicity categories (non-Hispanic Whites, non-Hispanic African Americans, non-Hispanic Others, Hispanics, Unknown), two categories for marital status (married vs. not married), and non-VA health insurance coverage (yes vs. no).

Provider-level covariates included provider type, as specialty clinicians are thought to be early adopters of telehealth [5, 16]. The GLA PC clinics are composed of PACT teams. The VA PACT teams have healthcare clinicians from different backgrounds including, physicians (MD), nurse practitioners (NP), Physician Assistants (PA), registered nurse (RN) care managers, licensed vocational nurses (LVN), pharmacists (Pharm), medical assistants (MSA) (i.e., clerks and schedulers), nutritionists/dieticians (DT), social workers (SW), and mental health providers (MH). For this study, type of PC clinicians was grouped in four categories: 1 = MD, NP, PA; 2 = LVN, MA; 3 = RN; 4 = SW, Pharm, DT; 5 = MH. Site-level covariates included site type to account for known rural versus urban disparities in telehealth use [11, 12]. GLA includes a main medical facility in West Los Angeles (WLA) and several community-based clinics throughout the greater Los Angeles area and surrounding counties. Therefore, site type was categorized WLA vs. community-based clinics.

### Statistical analyses

Individual-level interrupted time series (ITS) analysis through segmented logistic regression on repeated monthly observations of telehealth use over 24-months (March 1, 2019, through March 1, 2021) was used to test the impacts of COVID onset, re-expansion of in-person services at GLA and the start of the 2020 flu season on telehealth (or video) use. The time series was divided into four segments: 1) pre-COVID (March 2019 through February 2020), 2) onset of COVID-19 (stay-at-home orders were initiated March 2020 through May 2020), 3) the re-expansion of in-person services at GLA (June 2020 through October 2020), and 4) start of 2020 flu season (November 2020 through March 2021).

Multivariate associations of key patient demographic and clinical variables, as well as provider and site characteristics, with telehealth (or video) use were tested using logistic regressions for each dependent variable. The model specification included all predictor variables listed above, and a sex-by-age interaction term to examine whether younger women were more likely to use telehealth services compared to younger men. The associations of each categorical variable with telehealth (or video) use are expressed as differences in the adjusted percentages of telehealth (or video) use between each category and a reference category. For provider type, the first group of clinicians (i.e., MDs, NPs, PAs), and for the site variable, community-based clinics were set as the reference categories. The statistical significance level was set at *p* < 0.05. Analyses were conducted in Stata (version 17.0).

## Results

Table 1 compares patient characteristics of GLA PC patients, pre-COVID-19 (March 1, 2019-February 28, 2020) vs. during COVID-19 (March 1, 2020, through February 28, 2021). The results indicate very similar characteristics between the two groups of patients (pre- and post-pandemic), except for telehealth use. Before onset of COVID-19, only 13.9% of GLA PC patients used any telehealth services, where only 0.3% used video-based care. During the first 12-months of COVID-19, however, 63.1% of GLA PC patients used telehealth services and 11.3% used video-based care.

**Table 1:** Patient characteristics at the VA Greater Los Angeles Primary Care Clinics, 12-months before (March 1, 2019-February 28, 2020) and 12-months after onset of COVID-19 (March 1, 2020-February 28, 2021)

Patient Characteristics	12-months before onset of COVID-19 (*n* = 64,361)	12-months after onset of COVID-19 (*n* = 48,729)
Used Telehealth Services (phone or video)	13.9%	63.1%
Video Only	0.3%	11.3%
Male	91.4%	91.5%
Race:		
Non-Hispanic White	42.9%	43.1%
Non-Hispanic African American	20.9%	21.2%
Hispanic	17.7%	17.8%
Non-Hispanic Other	6.1%	5.9%
Unknown	12.5%	12.2%
Age Categories:		
18–44	23.1%	19.9%
45–64	27.6%	28.1%
65–74	28.9%	31.2%
75+	20.4%	20.8%
Married	40.3%	40.3%
Other Health Insurance	43.6%	46.0%
Main Medical Facility	24.0%	25.5%
Mean Age	60.5 (SD = 17.6)	61.7 (SD = 16.8)
Mean Nosos^a^	1.04 (SD = 1.40) (*n* = 61,914)	1.08 (SD = 1.41) (*n* = 47,248)

^a^Nosos is an indicator of comorbidities and health risk (see U.S. Department of Veterans Affairs. Risk Adjustment. 2016 Mar 16 [cited 2021 June 11]. Available from: http://vaww.herc.research.va.gov/include/page.asp?id=risk-adjustment#goes-into-nosos

**Fig. 1:**
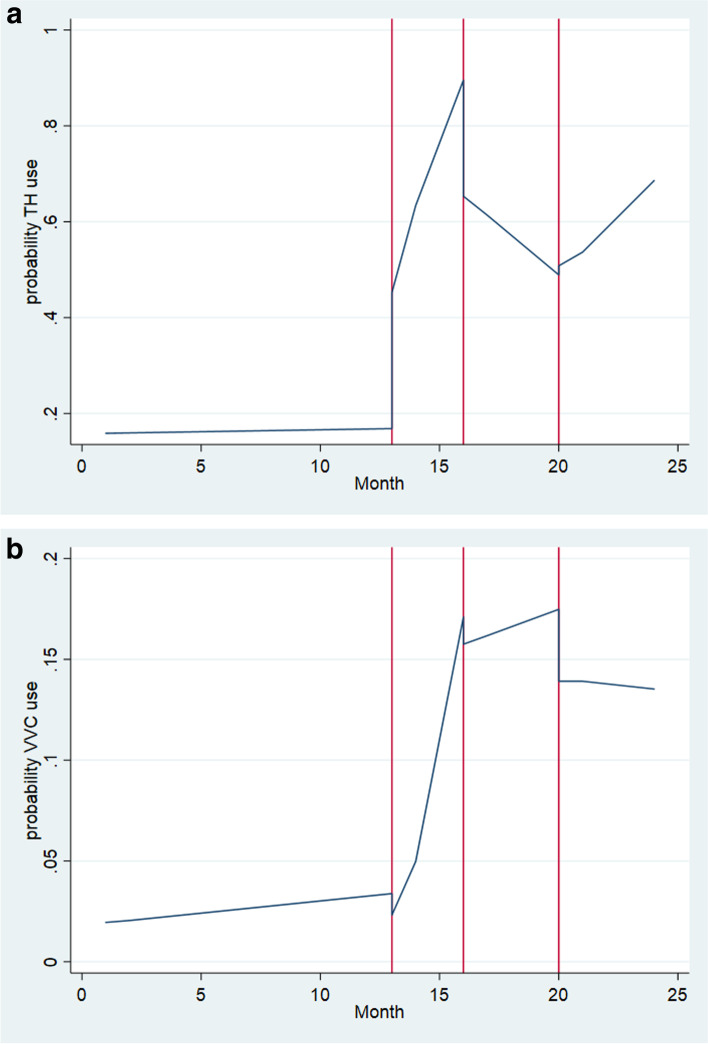
**a** For any virtual/telehealth (TH) use, in the pre-COVID segment, there was evidence of a slight increasing monthly trend in telehealth use. At the onset of COVID-19 (month 13, March 2020), rate of any telehealth use showed a highly significant, near-threefold increase of 28 percentage points (*p* < .001), and use showed significant monthly increases until the reauthorization of in-person services (month 16, June 2020). Rate of telehealth use then showed an immediate and significant dramatic reduction of 25 percentage points (*p* < .001) followed by a continued significant monthly reduction (*p* < .001). At the start of the 2020 flu season (month 20, November 2020), however, telehealth use showed a modest but significant increase followed by a significant monthly increase through the end of the time series. **b** For video-based care, however, different patterns of use emerged after the onset of COVID-19. Like telehealth use, before onset of COVID-19, there was evidence of an increasing monthly trend in video use (VVC). At the onset of COVID-19, however, there was a slight but significant decrease in video use, but this immediate reduction was followed by a sharp and highly significant increase until re-expansion of in-person services (month 16, June 2020), at which point video use had increased nearly eightfold. Video use then showed a slight immediate decrease, followed by a significant monthly increase until the start of the 2020 flu season (month 20, November 2020). Video use then showed a sharp and significant 20% decrease followed by a small but significant monthly decrease in video use until the end of the time series

Figure 1a illustrates the adjusted probabilities of any telehealth use over four-time segments: pre-COVID, onset of COVID-19, re-expansion of in-person services at GLA, and start of the 2020 flu season. Figure 1b illustrates the adjusted probabilities of video use by the same four-time segments. Both figures display the results of the shifts to intercepts and slopes following COVID-19 onset, in-person service re-expansion, and flu season onset.

The results of the logistic regression analysis are summarized in Table 2. These results illustrate adjusted percentages for site, provider, and patient predictors of any telehealth use (first column) and video use (second column) in PC at GLA. The findings indicate that there is evidence of adjusted associations for each patient, provider, and site-level characteristic with telehealth use, as well as video use. For site characteristics, Veterans at the GLA community-based clinics had a higher percentage of any telehealth use (37.7%) compared to Veterans receiving primary care at the main WLA medical facility (29.8%) (*p* < .001). Conversely, video use was lower among community-based clinics PC patients (9.5%) compared to PC patients at the WLA medical facility (13.1%) (*p* < .001).

**Table 2:** Patient, provider, and site characteristics of any virtual care or video-based care use at the VA Greater Los Angeles PC Clinics

Adjusted %^a^ [non-reference vs. reference (ref) difference in % any virtual care or video-based care (95% Confidence Interval) and *p*-value]				
Study Covariates	Any Virtual Care	*p*-value	Video-Based Care	*p*-value
Age & Sex Interaction:				
18–44 & Female (ref)	40.6% (N/A)	NA	20.2% (N/A)	NA
18–44 & Male	34.4% (−7.1, −5.2%)	<.001	15.3% (−6.0, −3.8%)	<.001
45–54 & Female (ref)	38.7% (N/A)	NA	17.2% (N/A)	NA
45–54 & Male	34.6% (−5.1, −3.2%)	<.001	10.6% (−7.6, − 5.6%)	<.001
65–74 & Female (ref)	37.8% N/A	NA	13.7% (N/A)	NA
65–74 & Male	34.9% (−4.5, −1.2%)	<.001	8.5% (−7.0, −3.5%)	<.001
75+ & Female (ref)	36.1% (N/A)	NA	7.2% (N/A)	NA
75+ & Male	35.4% (−3.1, 1.7%)	.555	7.0% (−2.0, 1.7%)	.854
Race/Ethnicity:				
Non-Hispanic White (ref)	35.3% (N/A)	NA	11.1% (N/A)	NA
Non-Hispanic African American	36.3% (0.5, 1.4%)	<.001	9.7% (−1.8, −1.0%)	<.001
Non-Hispanic All Other	34.7% (−1.3, 0.1%)	.100	11.5% (−0.3, 1.1%)	.300
Hispanic	34.4% (−1.3, − 0.4%)	<.001	10.2% (− 1.3, − 0.4%)	<.001
Unknown	34.6% (− 1.3, − 0.2%)	.011	10.2% (− 1.4, − 0.3%)	<.002
Marital Status:				
Not Married (ref)	35.0% (N/A)		9.6% (N/A)_	NA
Married	35.9% (0.6, 1.3%)	<.001	12.1% (2.1, 2.8%)	<.001
Non-VA Health Insurance:				
No (ref)	34.8% (N/A)	NA	10.0% (N/A)	NA
Yes	35.9% (0.7, 1.4%)	<.001	11.2% (0.9, 1.6%)	<.001
Health Risk Factors (Nosos)^b^:				
0.5 Nosos Value	35.1% (34.9, 35.3%)	<.001	11.3% (11.1, 11.5%)	<.001
1.0 Nosos Value	35.2% (35.0, 35.4%)	<.001	10.8% (10.6, 11.0%)	<.001
2.0 Nosos Value	35.4% (35.2, 35.6%)	<.001	9.8% (9.5, 10.0%)	<.001
PCClinician Type:				
MDs, PAs, NPs (ref)	34.0% (N/A)	NA	15.3% (N/A)	NA
LVNs, Medical Assistant (MAs)	24.7% (−9.6, −8.9%)	<.001	0.2% (−15.4, −14.8%)	<.001
Registered Nurse (RNs)	39.1% (4.8, 5.5%)	<.001	2.2% (−13.4, − 12.8%)	<.001
SWs, Pharmacists, Dietitians	53.7% (19.1, 20.3%)	<.001	5.4% (− 10.3, − 9.5%)	<.001
Mental Health Providers	41.3% (4.5, 10.0%)	<.001	43.3% (23.6, 32.4%)	<.001
Hospital-Based Clinic:				
No (e.g., community clinics) (ref)	37.7% (N/A)	NA	9.5% (N/A)	NA
Yes	29.8% (−8.2, −7.5%)	<.001	13.1% (3.2, 4.0%)	<.001

^a^Individual-level interrupted time series analysis using segmented logistic regression on repeated monthly observations over 24-months (March 1, 2019 through March 1, 2021) adjusting for patient and provider level clustering; patient sociodemographic variables, health status; provider and site characteristics

^b^The association of the continuous Nosos score with telehealth (or video) use is expressed as the adjusted percentage of telehealth (or video) use obtained from a Nosos value of 2.0 (indicating comorbidities whose costs are likely to be two times as high as the national average, or “high-cost” users), and a Nosos value of 0.5 (indicating comorbidities whose costs are likely to be only half the national average, or “low-cost” users)

Regarding provider type, social workers, pharmacists, and dietitians had the highest percentage of telehealth use (53.7%) compared to PC clinicians (34.0%) (*p* < .001). For video-based care, however, mental health providers (43.3%) in PC clinics had the highest video use compared to PC clinicians (15.3%) (*p* < .001).

Regarding patient characteristics, for telehealth use as well as video-based care, the sex-by-age interaction results indicated that for all age groups, except for the oldest age group (75+), women were more likely to use telehealth (e.g., 18–44 years of age: women 40.6% vs. men 34.4%, *p* < .001) or video than men (e.g., 18–44 years of age: women: 20.2% vs. men: 15.3%, *p* < .001) (see Table 2). It should be noted that telehealth use among younger women was higher compared to younger men, but these sex differences diminished among older age groups (45–64 and 65–74). For video use, on the other hand, except for the oldest age group (75+), women of all ages were more likely to use video than men. In terms of racial/ethnic differences, non-Hispanic African Americans (36.3%) had higher percentage of any telehealth use, while Hispanics (34.4%) had lower percentage of telehealth use compared to non-Hispanic Whites (35.3%) (*p*’s < .001). For video use, however, both Hispanics (10.2%) and non-Hispanic African Americans (9.7%) had a lower percentage compared to non-Hispanic Whites (11.1%) (*p*’s < .001). Similarly, use of telehealth services among those who have non-VA health insurance was higher (35.9% vs. 34.8%, *p* < .001) than among Veterans with only VA insurance. We saw the same pattern for video use (11.2% vs. 10.0%, respectively, *p* < .001). Regarding health factors, Veterans with higher Nosos scores had higher percentages of any telehealth use (Nosos score 0.5 vs. 2.0: 35.1% and.35.4%, respectively, *p* < .001), but for video use, Veterans with higher Nosos scores had lower percentages of video use (0.5 vs. 2.0: 11.3% and. 9.8%, respectively, *p* < .001).

## Discussion

Before the COVID-19 pandemic, the literature on telehealth [30–43] mainly focused on non-emergency situations, where patients and clinicians had a choice between virtual and in-person visits. Even with pre-COVID-19 telehealth research during major disasters, such as Hurricanes Sandy (in 2012), Harvey (in 2017), and Irma/Maria (in 2017), where there were disruptions in accessing in-person care, use of telehealth visits were limited and predominantly phone based [44–46]. However, with the onset of the pandemic, fear of infection quickly became a priority to protect all healthcare professionals and patients, which combined with organizational factors propelled the widespread and rapid expansion of real-time video telehealth to patients’ homes nationwide [1, 2, 4]. As a result, there was a dramatic shift to use of virtual care for outpatient services in PC and other healthcare services [5–8, 11, 20–27].

While several studies have examined patient (and some site) characteristics associated with telehealth use [6, 11], none have examined the simultaneous effect of multi-level determinants (including provider characteristics) of telehealth use immediately after the onset of COVID-19. In this study, we simultaneously examined site, provider, and patient characteristics associated with telehealth and video use in PC and confirmed that racial/ethnic minority groups were more likely to use telephone than video, consistent with both VA and non-VA research [1, 6, 41, 47]. Although studies on inequities regarding access to virtual care are inconsistent [48–50], major gaps persist in access to cellular data, video-capability, computer devices, adequate/high speed internet coverage and connectivity, which cannot be ignored when examining utilization of virtual care for all patient populations [13]. One component of addressing the racial/ethnic variations involves tackling the digital divide [51], as select groups, such as older individuals, and those living in rural areas, as well as individuals with socioeconomically disadvantaged backgrounds, may have more limited access to the internet or camera-enabled devices [51–53]. Since 2016, the VA has provided tablets/iPads to qualified Veterans, in order to address the digital divide [51–53], but additional research is still needed to have a better understanding of telehealth access points in rural areas with poor broadband (https://vaww.telehealth.va.gov/pgm/atlas/index.asp). Furthermore, the adoption, and/or expansion, of video-based care delivery depends on several other factors that are beyond the scope of this paper and require more research.

With respect to sex differences, the results from the sex and age interaction test illustrate a statistically significant interaction effect, where telehealth use was higher among younger women Veterans. This might allude to the fact that for women with additional familial responsibilities, such as childcare and caregiving to the elderly, telehealth might be a more viable option for this specific demographic. Although the lower telehealth or video use among older patients is not a new finding [54], the significant interaction effect between sex and age on telehealth use is novel, and more research is needed to better understand why younger women are more likely to use telehealth services compared to younger men.

Beyond patient characteristics, one key finding is the significant variation in site characteristics. Even though there are no studies that have studied PC telehealth use in VA medical centers compared to surrounding community-based clinics, our study finding, which indicates that Veterans at GLA community-based clinics were more likely to use telephone and less likely to use video use compared to Veterans receiving care at the main WLA medical facility, essentially alludes to what is known about rural vs. urban telehealth disparities inside and outside of the VA [55–60]. Furthermore, site variations in access to video care might be due to various factors, such as differences in bandwidth availability, clinic infrastructure, leadership buy-in, provider trainings, resources, camera-devices, and IT support. Site differences might also allude to the different types of PC services that are offered at less urban community clinics versus urban/main medical facilities.

Another key factor associated with telehealth use is type of provider. The study results indicate, compared to PCPs, social workers, nutritionists, and pharmacists were more likely to use any virtual care (mostly telephone), whereas mental health care providers were more likely to provide video-based care. Previous research has shown that mental health providers have a long tradition of using video visits, which may help explain the study findings [61, 62]. Furthermore, social workers have a long history of conducting case management, which might explain their preference for using telephone vs. video. Similarly, for pharmacists and dietitians, a telephone visit might suffice, when trying to connect with patients virtually especially for education or management type encounters. In sum, these differences might allude to the type of care that is being provided by the different types of providers in PACT teams – where some PC services might be more appropriate for telephone visits, whereas others might be more appropriate for video visits. Therefore, additional research is needed to identify which PC services are better suited for telephone- vs. vide-based care from both the provider’s as well as the patient’s perspectives. Moreover, more studies are needed to address how different types of outpatient clinics, such as community based and main medical facilities that have interdisciplinary teams of providers, can increase access to virtual care for all patient populations and different PC services.

## Limitations

This study has several limitations. First, this study is based on one VA medical facility and surrounding large and small community clinics that are in an urban/suburban area, which limits the generalizability of study findings to other VA facilities. Moreover, the patient population served by GLA, and surrounding community clinics might differ from other areas in the country, which further limits generalizability. However, it should be noted that GLA is the second largest healthcare system in the country with diverse patient populations, which provides a larger context of telehealth use. Another limitation is that studying telehealth use within VA may not be generalizable to other non-VA healthcare systems. However, VA is a leader in provision of telehealth services, therefore, many learned lessons might still be applicable to non-VA facilities. Furthermore, the study did not have access to other provider variables, such as provider’s age or provider’s comfort with telehealth, which might influence telehealth use or not use. These findings, however, do suggest a critical need for additional information about site-level and provider-level variations in use of virtual services, understanding variations in phone and/or video use between main VA medical facilities and community-based outpatient clinics, and what resources are needed to increase access to virtual care among all PC clinicians. With regards to the study design, it should be noted that the ITS design is not a true experimental design, but a quasi-experimental design, which requires multiple measurements of dependent variables both before and after the occurrence of the hypothesized causal event. A rule of thumb is eight observations both before and after the event. There is also no control population in the basic ITS design, although a control time series can be included under some conditions [63]. Additionally, given that we did not have access to the number of COVID-19 cases, we were unable to provide any information on COVID-19 cases and telehealth use. Finally, given that many factors, such as COVID-19 infection rates among patients and providers, impacted the rapid expansion and use of telehealth outpatient services, it is difficult to precisely disentangle the impact of the various contextual factors on telehealth use.

## Conclusion

The study findings indicate that the determinants of telehealth (or video) use are multifaceted, yet different depending on the type of telehealth modality. Site, provider, and patient characteristics should all be considered to improve access to virtual care for all patient populations at the VA. Moreover, since telehealth care provision is likely to continue after COVID-19, additional research is needed to identify which PC outpatient services are better suited for telephone (e.g., case management) versus video-based care (e.g., integrated mental health visits). Furthermore, given that remote care access is increasingly common, there are still many unanswered questions about tele-consultation quality and patient outcomes. In sum, it is important to understand how all clinics can systematically increase access to both telephone- and video-based PC services, while ensuring equity care for all patient populations.

## Data Availability

The datasets generated and/or analyzed during the current study are not publicly available because it contains identifiable information.

## Abbreviations

PC: Primary Care
VA: US Veterans Health Administration
VVC: VA Video Connect
PACT: Patient-Aligned Care Teams
HBPC: Home-Based Primary Care
GLA: VA Greater Los Angeles Healthcare System
MD: Physicians
NP: Nurse Practitioners
PA: Physician Assistants
RN: Registered Nurse
LVN: Licensed Vocational Nurses
Pharm: Pharmacists
MSA: Medical Assistants
DT: Nutritionists/Dieticians
SW: Social Workers
MH: Mental Health
WLA: West Los Angeles
ITS: Individual-level Interrupted Time Series
